# Lessons from the Mainland of China’s Epidemic Experience in the First Phase about the Growth Rules of Infected and Recovered Cases of COVID-19 Worldwide

**DOI:** 10.1007/s13753-020-00294-7

**Published:** 2020-08-18

**Authors:** Chuanliang Han, Yimeng Liu, Jiting Tang, Yuyao Zhu, Carlo Jaeger, Saini Yang

**Affiliations:** 1 State Key Laboratory of Cognitive Neuroscience and Learning and IDG/McGovern Institute for Brain Research, Beijing Normal University, Beijing, 100875 China; 2 Key Laboratory of Environmental Change and Natural Disaster, Ministry of Education, Beijing Normal University, Beijing, 100875 China; 3 State Key Laboratory of Earth Surface Processes and Resource Ecology, Beijing Normal University, Beijing, 100875 China; 4 Academy of Disaster Reduction and Emergency Management, Ministry of Emergency Management and Ministry of Education, Faculty of Geographical Science, Beijing Normal University, Beijing, 100875 China; 5 Global Climate Forum, 10178 Berlin, Germany

**Keywords:** China, COVID-2019, Epidemiological properties, Logistic model, Pandemic

## Abstract

The first phase of the novel coronavirus disease (COVID-19) that emerged at the end of 2019 has been brought under control in the mainland of China in March, while it is still spreading globally. When the pandemic will end is a question of great concern. A logistic model that depicts the growth rules of infected and recovered cases in China’s mainland may shed some light on this question. This model well explained the data by 13 April from 31 countries that have been experiencing serious COVID-2019 outbreaks (*R*^2^ ≥ 0.95). Based on this model, the semi-saturation period (SSP) of infected cases in those countries ranges from 3 March to 18 June. According to the linear relationship between the growth rules for infected and for recovered cases identified from the Chinese data, we predicted that the SSP of the recovered cases outside China ranges from 22 March to 8 July. More importantly, we found a strong positive correlation between the SSP of infected cases and the timing of a government’s response. Finally, this model was also applied to four regions that went through other coronavirus or Ebola virus epidemics (*R*^2^ ≥ 0.95). There is a negative correlation between the death rate and the logistic growth rate. These findings provide strong evidence for the effectiveness of rapid epidemic control measures in various countries.

## Introduction

In December 2019, a series of cases of pneumonia with unknown cause was reported in Wuhan, the capital of Hubei Province in China (WHO 2020a). Later, deep-sequencing analysis of lower respiratory samples confirmed the presence of a novel coronavirus that may have originated from certain bats (Zhou et al. 2020), and was first named the 2019 novel coronavirus (2019-nCoV) on 12 January 2020. The coronavirus was officially renamed SARS-CoV-2 by the Coronavirus Study Group of the International Committee on Taxonomy of Viruses, and on 12 February 2020 the World Health Organization (WHO) named the disease caused by that virus COVID-19 (often this label is also used to refer to the virus). Human-to-human transmission of COVID-19 has been confirmed not only in China (Hui et al. 2020; Li et al. 2020; Wang, Hu, et al. 2020; Zhu et al. 2020), but in countries around the world (Roberts 2020)—such as the Republic of Korea (Choi and Ki 2020; Ki and Task Force for 2019-nCov 2020; Shim et al. 2020); Italy (Livingston and Bucher 2020; Rosenbaum 2020; Spina et al. 2020); and Iran (Abdi 2020; Tuite et al. 2020; Zhuang et al. 2020). We defined the first phase of the epidemic situation in China as from the beginning of January to early March, and the increase in the number of infections caused by overseas imports is the second phase, which started from mid-March.

Since March the first stage of epidemic situation in China’s mainland has been effectively controlled; there have been only sparse daily local new cases since 19 March 2020, and the recovery rate in China’s mainland had risen to 94.52% by 13 April (CCDC 2020a). However, the epidemic situation outside China is worsening. In the first phase of the epidemic, as of 13 April 2020, globally, a total of 1,788,665 confirmed cases of COVID-19, and death and recovered rates of 6.35% and 20.45%, respectively, were reported (Sina 2020). When the inflection point will appear for both infection and recovery outside China remains unclear. The hope for and lessons on COVID-19 control from China are necessary and worth quantifying (Azman and Luquero 2020).

We applied a descriptive model^1^ to analyze the global data on COVID-19 dynamics (infected and recovered cases in China’s mainland, and infected cases in countries outside China) by 13 April. We then estimated the relation between the parameters of infected and recovered cases in China’s mainland. Next, we used that relationship to map the parameter space of infected cases to the parameter space of recovered cases for 31 countries globally. Finally, we explored the relationship between the model parameters and governmental control measures.

## Method

This article proposes a simple logistic model to describe the epidemic of COVID-19 using public data.

### Data Sources

The cumulative number of confirmed and of recovered COVID-19 cases in China’s mainland, for the period from 10 January to 13 April 2020, was obtained from the National Health Commission of China and the Provincial Health Commissions of 30 provincial administrative regions (excluding Tibet, because the only confirmed infected COVID-19 case in Tibet was declared recovered on 12 February). The data are publically available. All cases were laboratory confirmed following the standards published by the National Health Commission of China (CCDC 2020b). The basic test procedure has been described in detail in previous work (Huang et al. 2020; Zhou et al. 2020). The acquired dataset was partially analyzed in an initial study on this topic.^1^ The data may involve systematic estimation errors because there was no way to identify all asymptomatic infections, and therefore no way to identify recoveries from such infections as well. However, as the relevant estimation errors have an understandable systemic character, the growth rates between data from different days are reliable. Our analysis works exclusively with these growth rates.

For our subsequent analysis, we selected 31 countries outside China with serious coronavirus epidemic situations where data were available for the period from 10 January to 13 April 2020. Their population amounts to 39.4% of the world population (57.8% with China included) and 81.6% of the infected cases documented globally (86.2% with China included) (WHO 2020a). The data for COVID-19 cases in the 31 selected countries were obtained from the situation reports on the official website of the World Health Organization (WHO 2020a), which is publicly available. The data used in this study include the cumulative number of reported laboratory-confirmed COVID-19 cases. The countries (beyond China) included in our study are: Australia, Japan, Republic of Korea, and Singapore, in the western Pacific region; Austria, Belgium, Bulgaria, Czech Republic, Denmark, Finland, France, Germany, Italy, Lithuania, Portugal, Slovakia, Spain, Sweden, Switzerland, the Netherlands, the United Kingdom and Ukraine in Europe; India in Southeast Asia; the Islamic Republic of Iran and Lebanon, in the eastern Mediterranean region; Brazil, Canada, Mexico, and the United States of America, in the Americas; Ethiopia and South Africa in Africa. All the laboratory-confirmed cases are determined according to the WHO standard.

The severe acute respiratory syndrome (SARS) in 2003 was another fatal coronavirus epidemic over the last two decades (Hung et al. 2018). We collected the SARS data (the cumulative number of suspected and dead cases), for the period 1 March to 16 August 2003, in Beijing from the National Health and Family Planning Commission of the People’s Republic of China (renamed as Chinese Center for Disease Control and Prevention) (CCDC 2020c). The Middle East respiratory syndrome (MERS) in 2012 was a coronavirus epidemic in more recent years. We collected the MERS data (the cumulative number of suspected and dead cases), for the period 24 June 2012 to 3 June 2016, in Saudi Arabia from Saudi Arabia’s Ministry of Health (MOH) (Saudi Arabia’s Ministry of Health 2020). All these data are publicly available.

We collected the Ebola data (the cumulative number of suspected and dead cases), for the period 23 March 2014 to 30 March 2016, in Guinea and Sierra Leone from the reports on the official website of the World Health Organization (WHO 2020b).

The governments of the 31 countries selected for this study all declared a state of national emergency, or wartime, or a closing of their borders as a result of the COVID-19 outbreak. We collected data on the beginning of government intervention from the mainstream authoritative media of each country selected.

### Epidemic Curve Modeling

Previous studies have shown that the daily cumulative number of SARS patients could be explained by a logistic function (Huang et al. 2003; Wang and Liu 2005). Based on this assumption, we modeled the epidemic information of the virus infection cases in logistic form (Eq. 1):

1 $$\left\{ {\begin{array}{*{20}c} {\frac{dI\left( t \right)}{dt} = \alpha I\left( t \right)\left( {1 - \frac{I\left( t \right)}{{N_{1} }}} \right)} \\ {I\left( 0 \right) = I_{0} } \\ \end{array} } \right.$$

where *N*_1_ is the maximum number of cumulative infections, *I*(*t*) is the cumulative number of patients at time *t*, $\alpha$ is the logistic growth rate, and *I*_0_ is the number of infected cases at the initial time.

The analytical solution of Eq. 1 could be written in the form of Eqs. 2 and 3:

2 $$I\left( t \right) = \frac{{N_{1} }}{{1 + C_{1} e^{ - \alpha t} }}$$

3 $$C_{1} = \frac{{N_{1} }}{{I_{0} }} - 1$$

where *C*_1_ is a constant term determined by the initial state.

Similarly, the analytical solutions for the recovered cases are represented as Eqs. 4 and 5:

4 $$R\left( t \right) = \frac{{N_{2} }}{{1 + C_{2} e^{ - \beta t} }}$$

5 $$C_{2} = \left( {\frac{{N_{2} }}{{R_{0} }} - 1} \right)$$

where $\beta$ is the logistic growth rate of recovered cases, *N*_2_ is the maximum number of cumulative recovered cases, and *R*_0_ is the number of recovered cases at the initial time, *C*_2_ is a constant term determined by the initial state.

Here we transformed Eqs. 2–4 into a more intuitive form and unified them as Eq. 6:

6 $$N\left( t \right) = \frac{A}{{1 + e^{{ - k\left( {t - t_{0} } \right)}} }}$$

where *N*(*t*) is the general form of the cumulative number of infected or recovered patients at time *t*, *A* denotes the maximum number of infections or recoveries, *k* is the logistic growth rate, and *t*_0_ is the semi-saturation period (SSP), that is, the inflection point of the sigmoid curve.

This descriptive model has been verified with the infected, death, and recovered cases of COVID-19,^1^ and also with the SARS data from 2003.^1^ For the infected cases, there are three parameters (*A*, *k*, *t*_0_) in the model; for the recovered cases, we fixed *A* to the difference between the maximum number of cumulative infections and deaths, based on biological fact. In our model, *t*_0_ is the mathematically defined inflection point. In this article, we assume that *t*_0_ is the time of inflection of the epidemic dynamics in a region.

We processed the data and modeled them with custom scripts on MATLAB (the Math Works). We adopted the nonlinear least squares (NLS) algorithm for data fitting and parameter estimation.

## Results

**Fig. 1:**
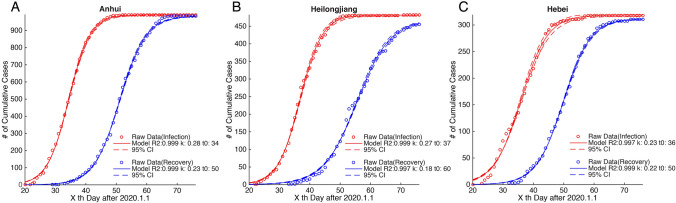
Example of provinces for the time series of COVID-19 infected and recovered cases with corresponding fitted curves in China’s mainland

Based on the cumulative number of confirmed and recovered cases in 30 provinces of China’s mainland as well as in 31 countries, with the above-mentioned model all data could be well explained (*R*^2^ > 0.95) (Figs. 1a–c, 5a–c, 11a–d).

### Relationship Between Infected and Recovered Cases in China’s Mainland

**Fig. 2:**
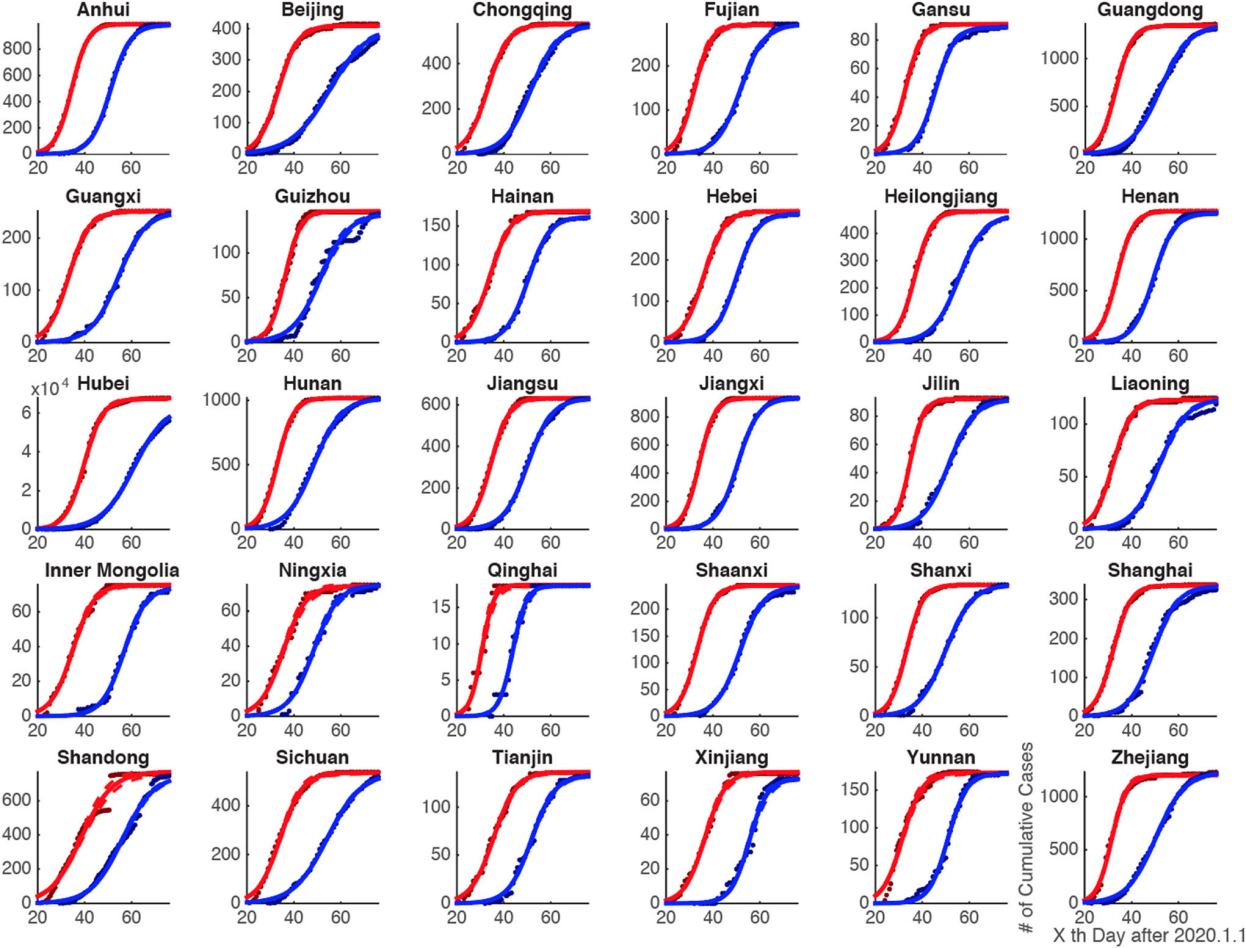
Intrinsic growth rules of patients infected with the 2019 novel coronavirus in 30 provinces of China

The first phase of epidemic situation of COVID-19 has been controlled in China, with a recovery rate of 94.52% by 13 April 2020 (CCDC 2020a). The time series of infected and recovered cases and their fitted curves for three example provinces (Anhui, Heilongjiang, and Hebei) are shown in Fig. 1a–c. By observing the fitted curves of the 30 provinces in China (Fig. 2), we found that the logistic growth rate (*k*) of infected cases is larger than that of recovered cases, and that the semi-saturation period (*t*_0_) of infected cases is shorter than that of recovered cases.

**Fig. 3:**
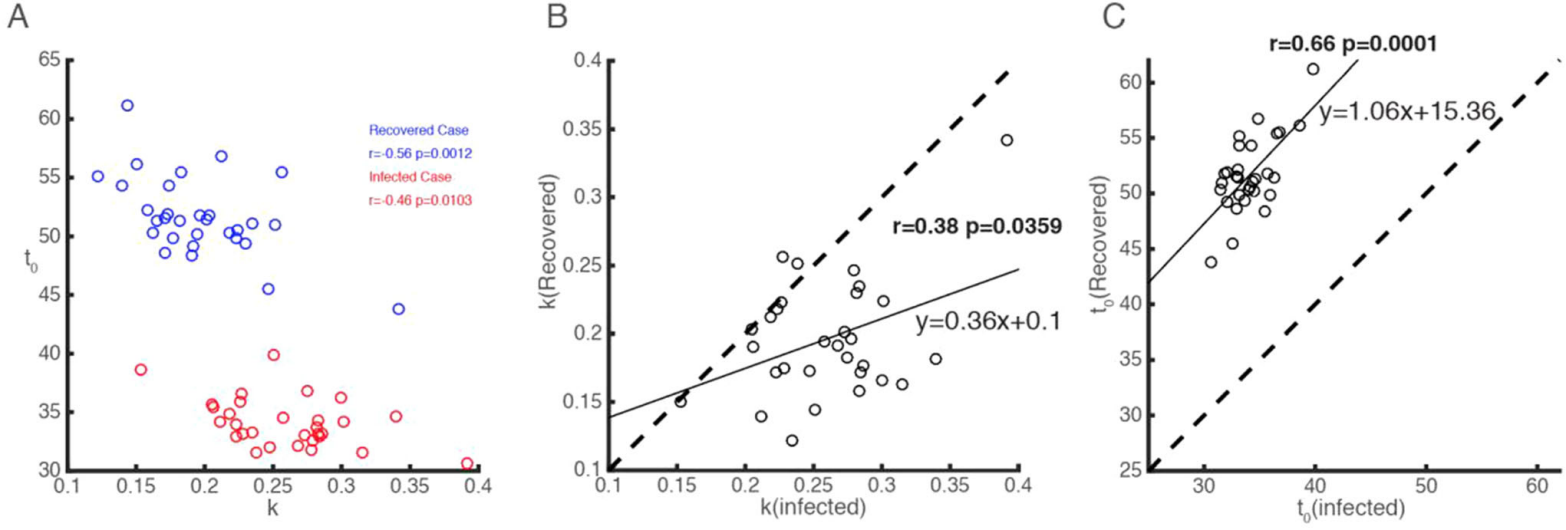
Relationship between the rules for COVID-19 infected and recovered cases in 30 provinces of China

Figure 3 shows that the parameter spaces of *k* and *t*_0_ are different for infected (red circles) and recovered cases (blue circles), and there is a significant negative correlation between the two (for infected cases, *r *= − 0.46, *p *= 0.0103; for recovered cases, *r *= − 0.56, *p *= 0.0012; Pearson correlation). When we compared these two parameters of infected and recovered cases, we found that infected cases have larger (*t *= 6.9136, *p *< 10^−7^, right-tailed test) growth rates (Fig. 3b) but smaller (*t *= − 37.271, *p *< 10^−25^, left-tailed test) semi-saturation periods (Fig. 3c) than recovered cases. The growth rates of infected and recovered cases are significantly positively correlated (*r *= 0.38, *p *= 0.0359, Pearson correlation), and so are the semi-saturation periods of infected and recovered cases (*r *= 0.66, *p *= 0.0001, Pearson correlation). The growth rates and SSPs of the provinces can be fitted to a linear model, with slope and intercept of 0.36 (CI: [0.0268, 0.698]) and 0.1 (CI: [0.014, 0.191]) for the growth rates (panel B), and slope and intercept of 1.06 (CI: [0.59, 1.54]) and 15.36 (CI: [− 0.85, 31.56]) for the SSPs (panel C).

### Growth Rules of COVID-19 Infections outside China

**Fig. 4:**
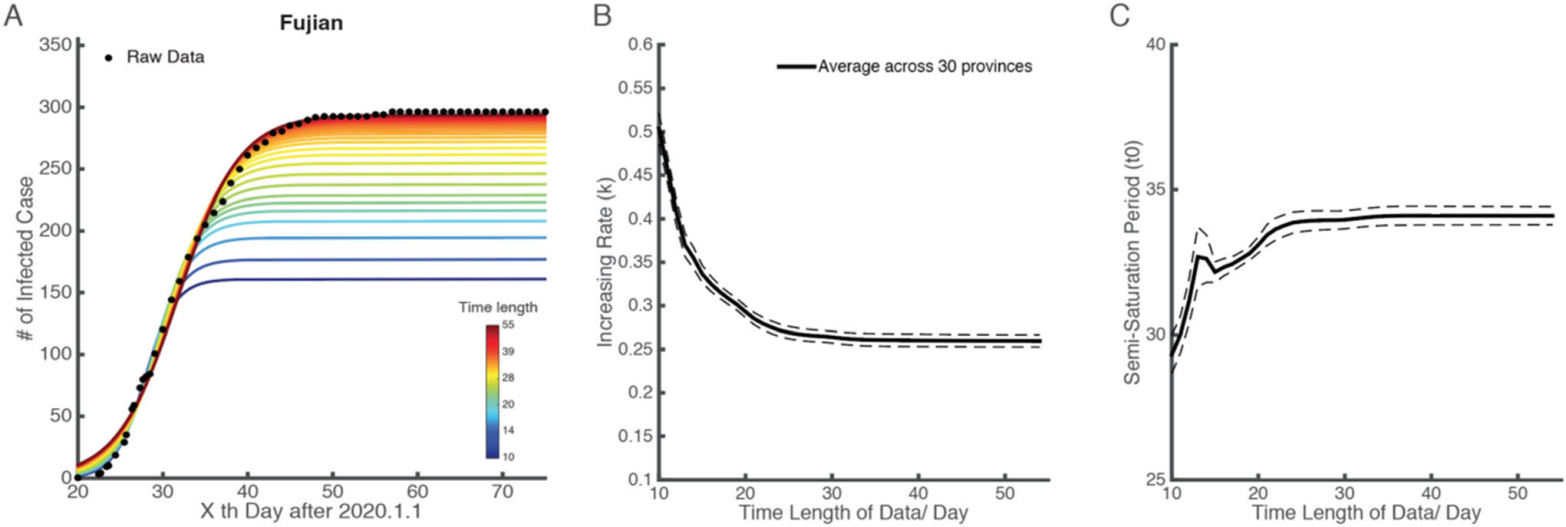
Time stability of the logistic model for COVID-19 infected cases in China’s mainland

The dataset of infected cases in China’s mainland shows the time stability of the logistic model (Fig. 4). The example of the province of Fujian is shown in Fig. 4a. As the length of the time series increases, the fitting curves converge to the actual number of cases (black dots).

We defined an index for minimum time length (MTL) to measure the amount of data necessary to produce a stable output. This index is the time length at which the change of parameter value (*k* or *t*_0_) is less than 5% in two consecutive days. With the data of 30 provinces in China, we found that the MTL for the logistic growth rate (*k*) is 15 days (Fig. 4b) and for the semi-saturation period (*t*_0_) it is 26 days (Fig. 4c). This result indicates the amount of data required to measure the epidemic situation.

**Fig. 5:**
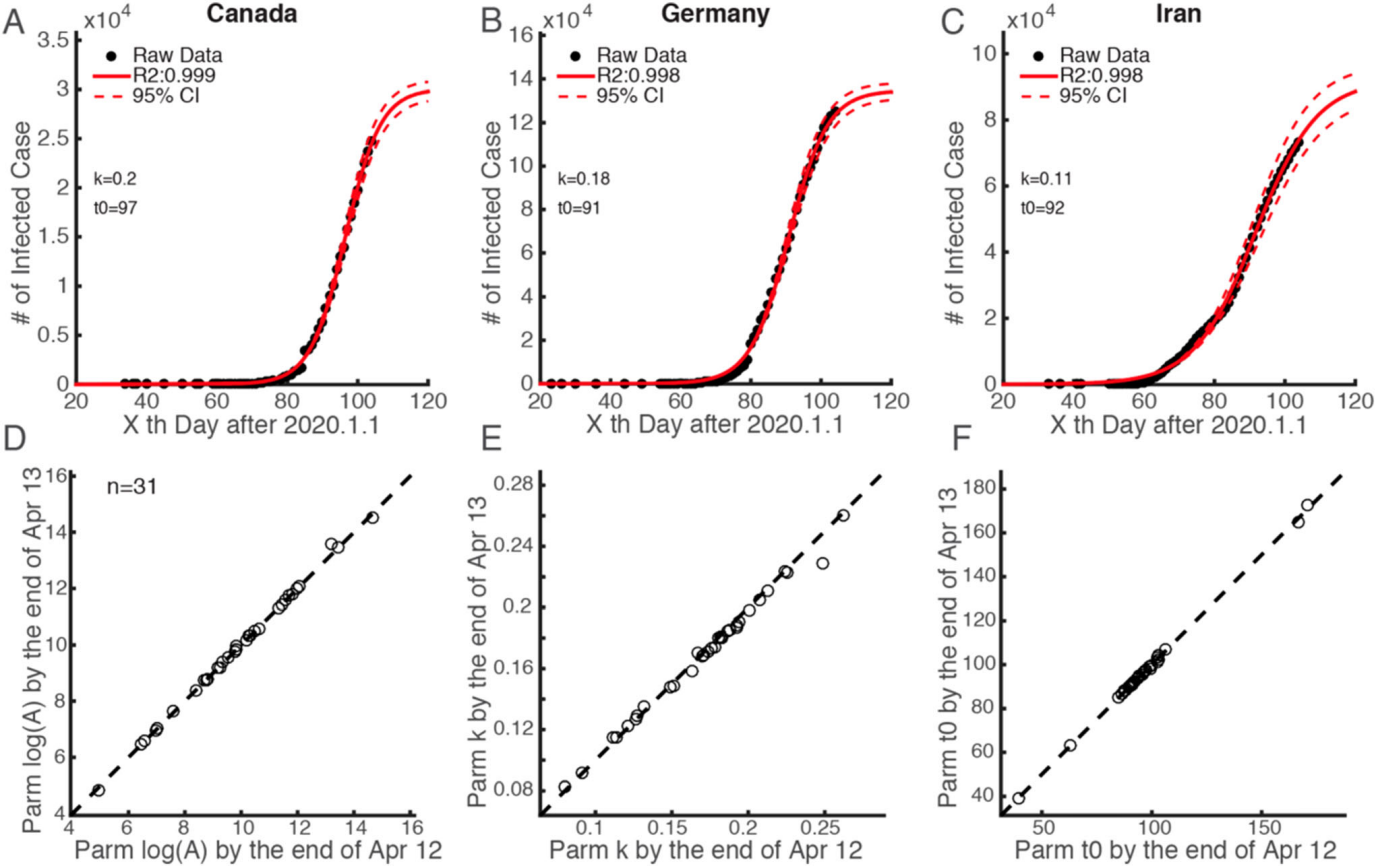
Example countries for the time series of COVID-19 infected cases with corresponding fitted curves around the world and their temporal stability

**Fig. 6:**
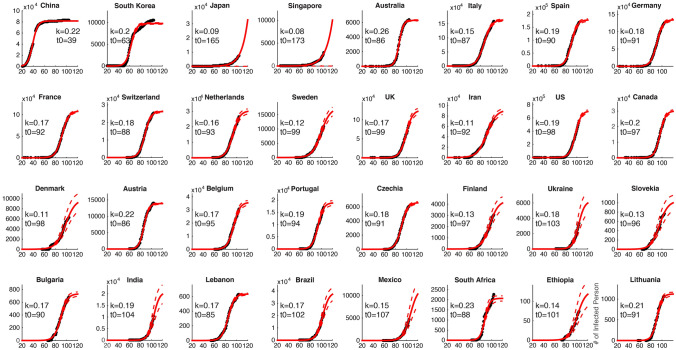
Intrinsic growth rules of patients infected with the 2019 novel coronavirus in China and 31 other countries around the world

After identifying the relationship between the increasing rate and the semi-saturation period, we applied this relationship to the COVID-19 cases from the selected 31 countries. We applied the descriptive model to fit the data for the cumulative number of infected cases in each of the 31 countries. Figure 5 shows the time series data of infection for three countries with the highest *R*^2^ (Canada, Germany, and Iran, *R*^2^ > 0.995; Fig. 5a–c). All data series for infected cases could be well explained by our model (*R*^2^ > 0.95) (see Fig. 6 for all 31 countries). The average change rates of the parameters are already lower than 5% (Fig. 5d–f), which indicates that the parameters we estimated from the model are stable. We noticed that the logistic growth rate of the 31 country cases does not show a significant difference (*t *= 1.197, *p *= 0.237 two-tailed test) from that of the Chinese provinces, but the 31country cases have a significantly longer (*t *= − 17.4819, *p *< 10^−20^, right-tailed test) semi-saturation period. The numbers indicate that the *t*_0_ of infections in the 31 countries has an average of 98 days (std: 20.5), ranging from 3 March to 18 June.

### Predictions for the Growth Rules of Recovered COVID-19 Cases and Their Relation to the Timing of Governmental Control Measures

**Fig. 7:**
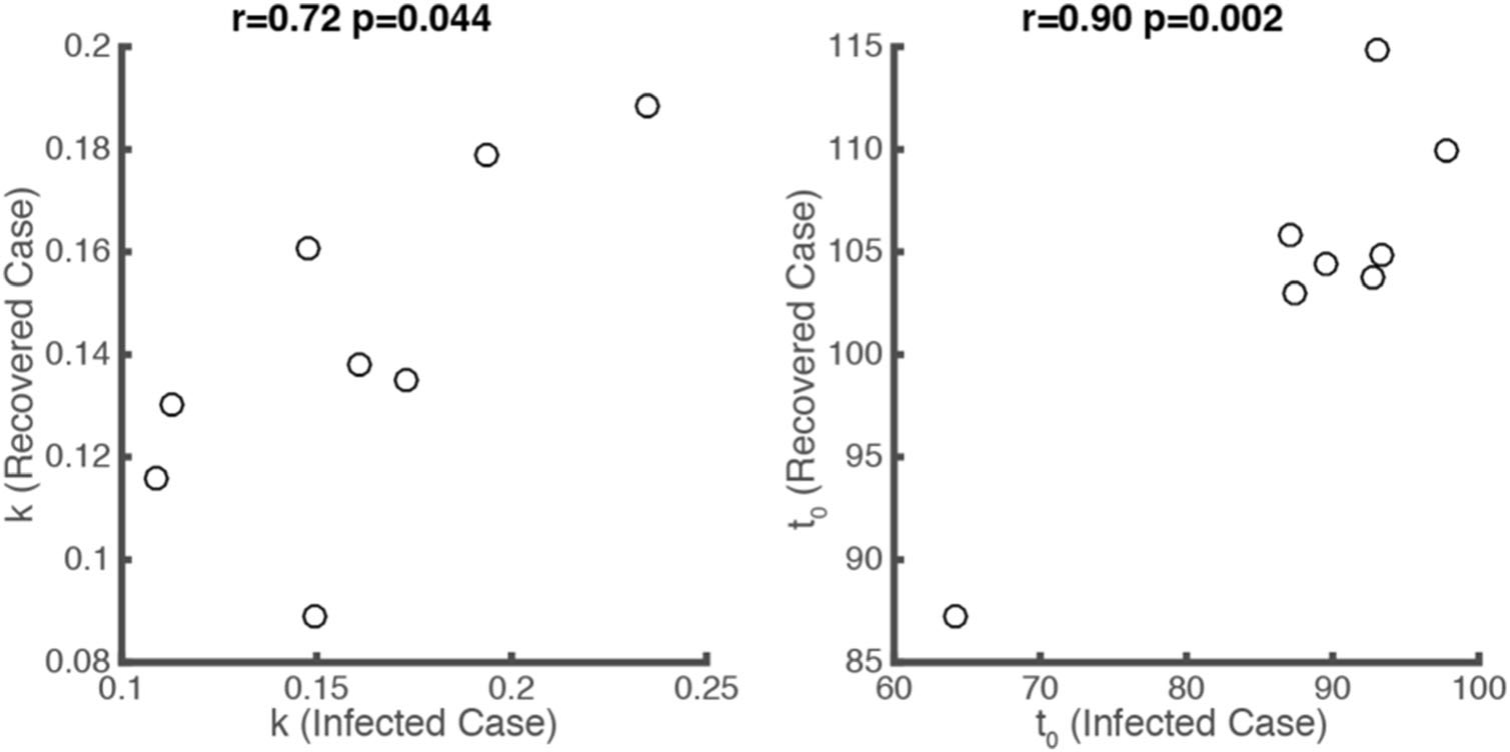
Relationship between the rules for infected and recovered COVID-19 cases in eight selected countries (Australia, Austria, Denmark, Germany, Iran, Republic of Korea, Spain, and Switzerland)

**Fig. 8:**
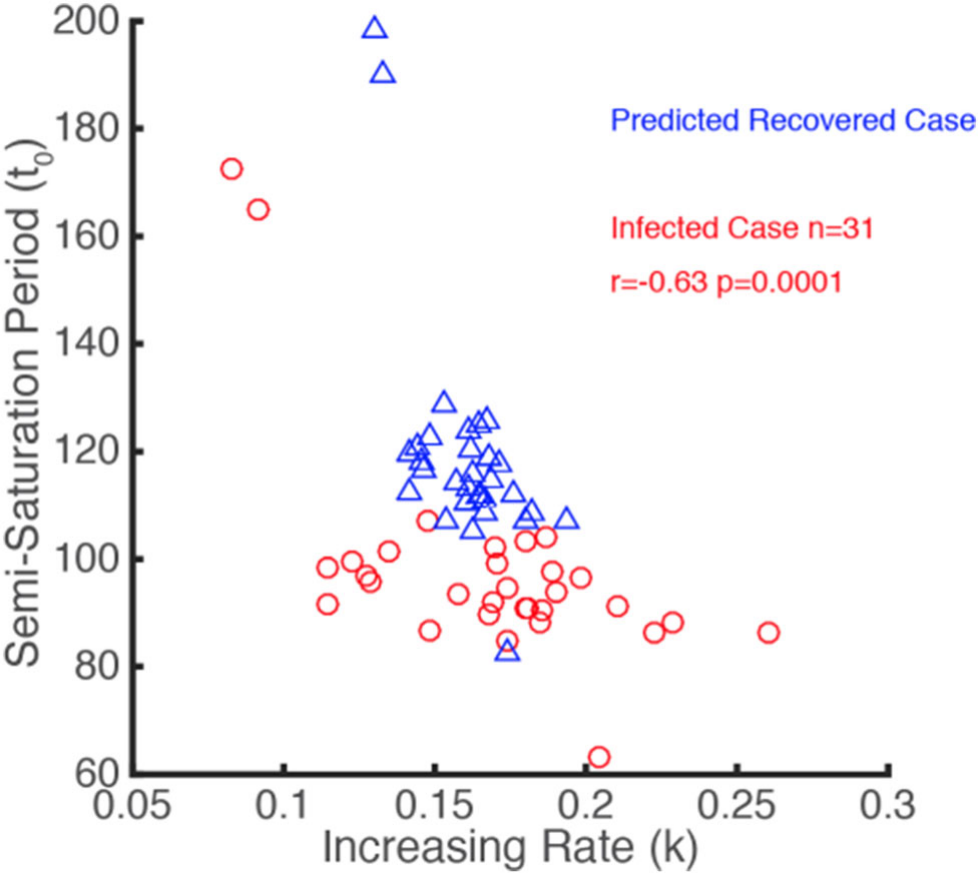
Prediction for growth rules of COVID-19 recovered cases around the world based on the Chinese experience

We found the same correlation as that in Fig. 3 for eight (Australia, Austria, Denmark, Germany, Iran, Republic of Korea, Spain, and Switzerland) out of the 31 countries globally with a recovery rate greater than 60% by 13 April (Fig. 7). On observing the logistic growth rate (*k*) and the semi-saturation period (*t*_0_) of infected cases in the selected 31 countries, we found there was a negative correlation (*r* = − 0.63, *p* = 0.0001, Pearson correlation) (Fig. 8) between these two parameters, which shows a similar tendency to the results of the 30 provinces in China (Fig. 3a). To predict the logistic growth rate (*k*) and the semi-saturation (that is, inflection) point (*t*_0_) of the recovered COVID-19 cases outside China, we mapped the parameter space of infected cases into the parameter space of recovered cases, based on the parameter relationship between infected cases and recovered cases obtained from China (Fig. 3b, c). For the 31 countries, the logistic growth rate ranges from 0.08 to 0.45. The mean *t*_0_ of the fitted curves for recovered cases is 119.71 (std: 21.73), which means that the mean semi-saturation period (*t*_0_) for these 31 countries arrived on, approximately 29 April 2020 (ranging from 22 March to 8 July).

**Fig. 9:**
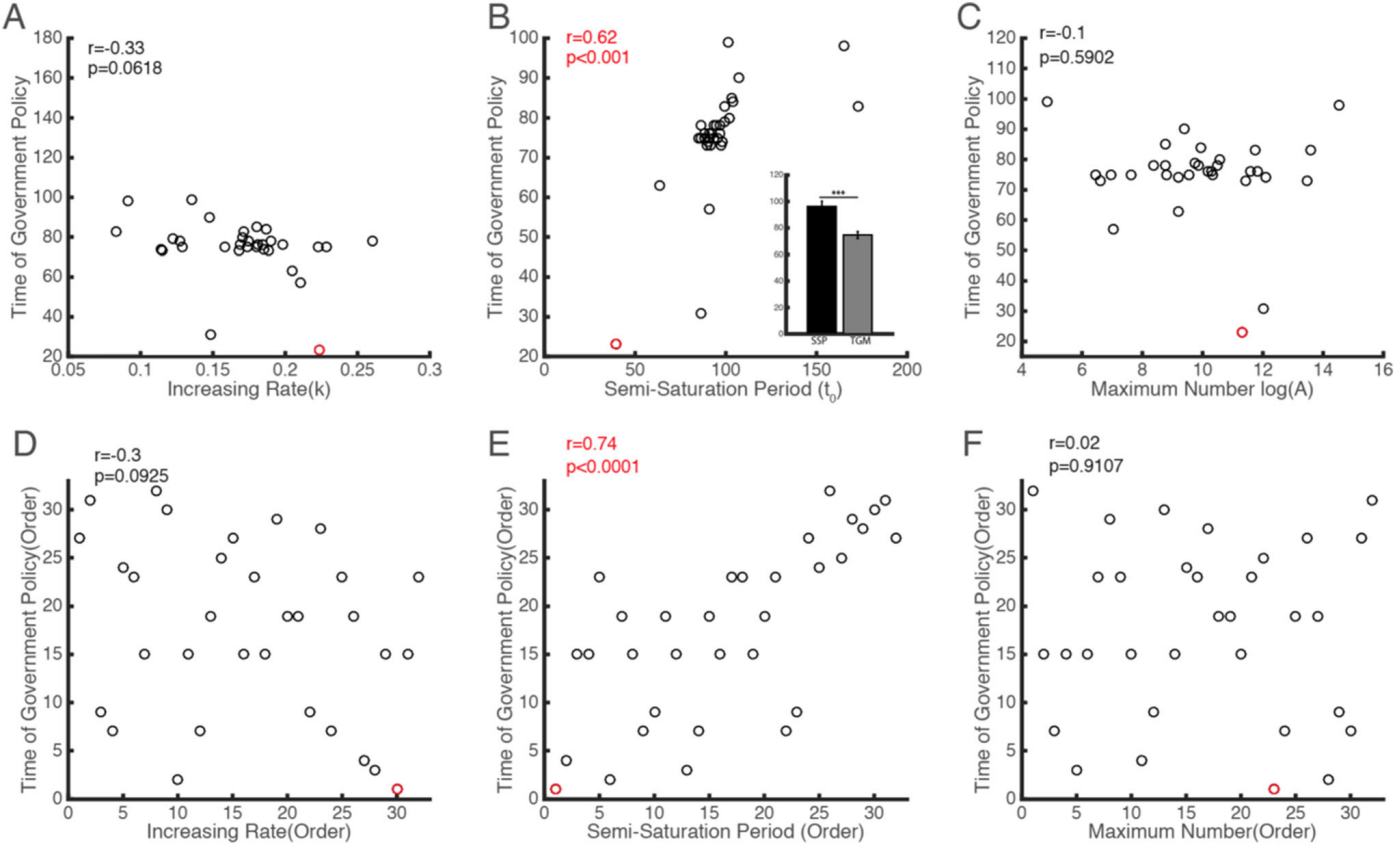
Relationship between the timing of government intervention and the model parameters of infected COVID-19 cases for each country

To explore the practical implication of these parameters, we took the timing of governmental emergency control measures related to COVID-19 as a variable to analyze its relationship with the key parameters (Fig. 9). We found that the semi-saturation time showed a significant positive correlation (*r *= 0.73, *p *< 0.0001, Spearman correlation) with the timing of governmental control measures (Fig. 9b, e).

**Fig. 10:**
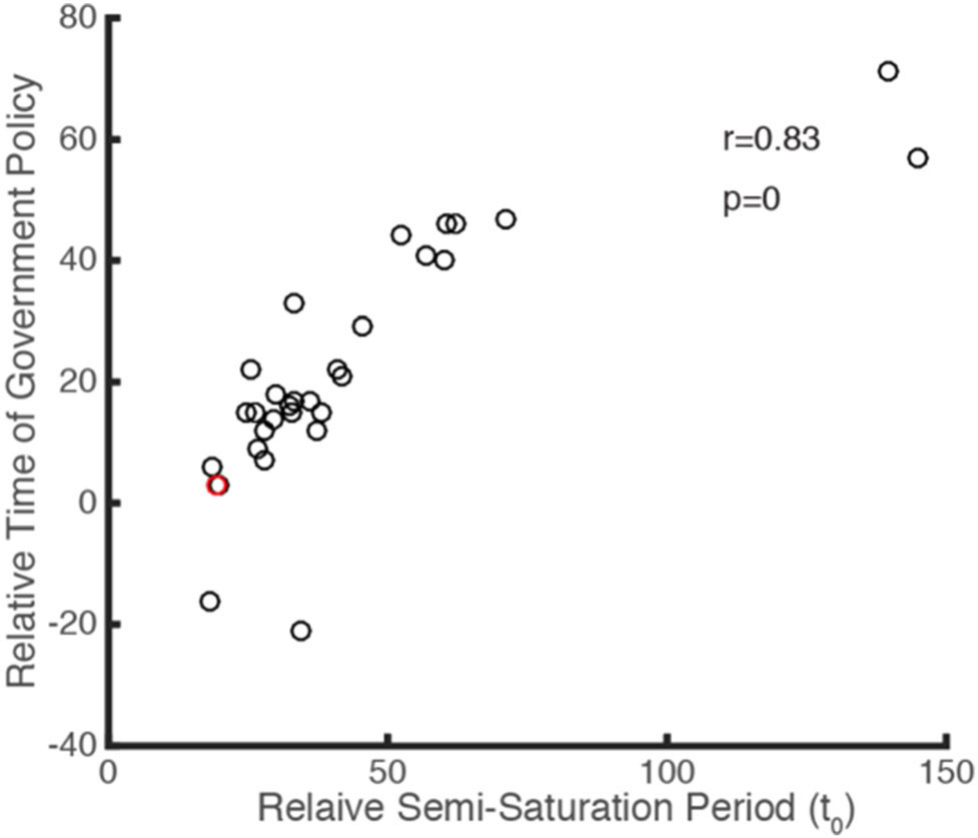
Relationship between the relative timing of government intervention and the semi-saturation period of infected COVID-19 cases for each country

In order to check this result more rigorously, we defined a relative semi-saturation time, calculating from the date with more than five infected cases in each country (rather than 1 January), and defined a relative timing of governmental control measures as the subtraction of absolute timing of government measures and the date of more than five infected cases in each country. We found the relative timing of governmental control measures is also positively correlated with the relative semi-saturation time (*r *= 0.83, *p *< 0.0001, Pearson correlation) (Fig. 10). The logistic growth rate showed a marginally significant negative correlation (*r *= − 0.3, *p *= 0.0925, Spearman correlation), while the maximum of infected cases did not show a strong correlation (*r *= 0.02, *p *= 0.91, Spearman correlation) with the timing of control measures (Figs. 9a–f). China released the earliest national control measures to prevent the spread of COVID-19 (on 23 January) and its *t*_0_ is the lowest (Fig. 9, red circle). We also noticed that the timing of governmental emergency control measures is significantly earlier than the semi-saturation period (Fig. 9b) (*t *= 2.99, *p *= 0.0043, two-tailed test), which indicates that most of the selected countries took measures in the early phase of the outbreak.

### Relationship Between Death Rate and *k*

**Fig. 11:**
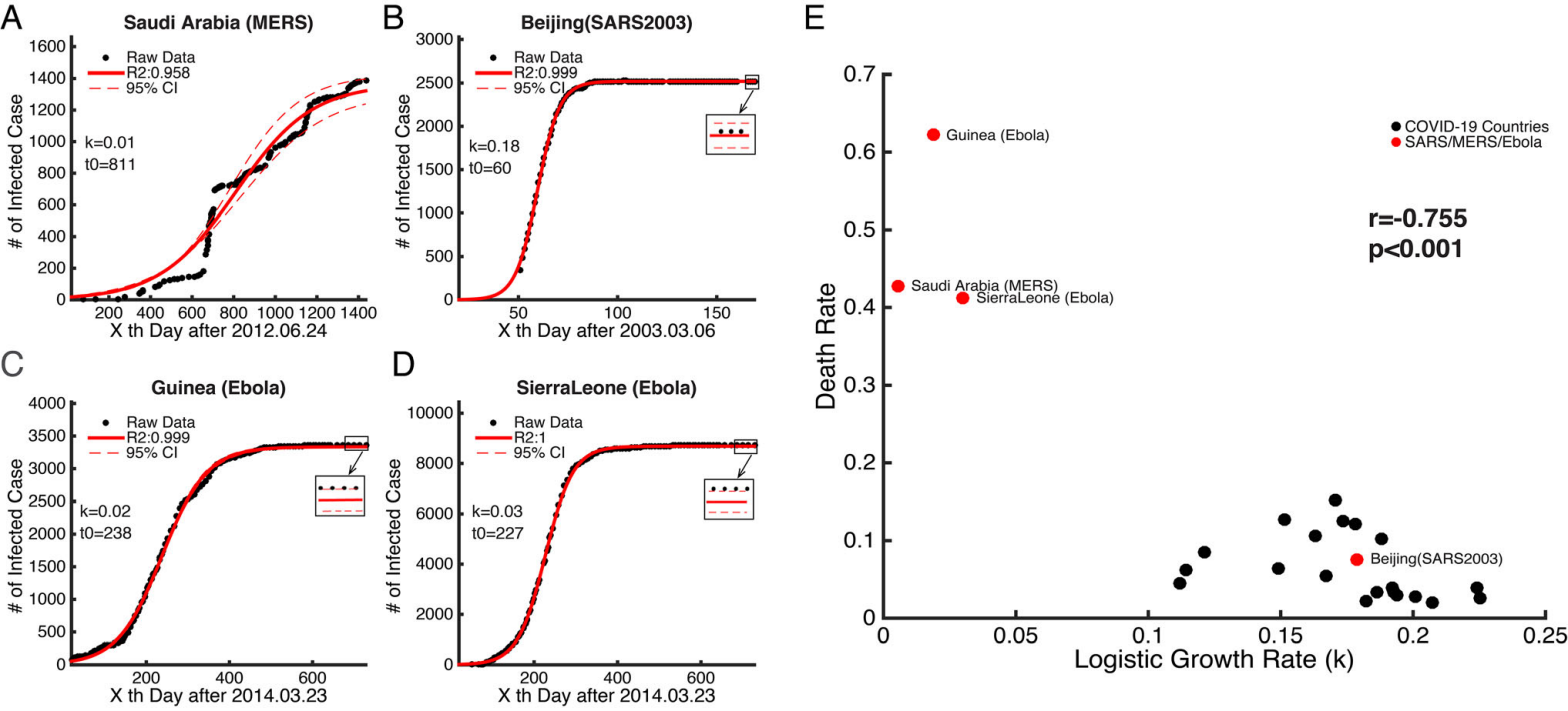
Example of the time series of cases infected with MERS, SARS, and Ebola, and the relationship between the death rate and the logistic growth rate (*k*)

Finally, this model was extended to four regions or countries (Saudi Arabia—MERS; Beijing—SARS; Guinea—Ebola; and Sierra Leone—Ebola) that experienced other coronavirus or Ebola virus epidemics before. Figure 11a–d show the time series data of infected cases and their fitted curves for these four regions or countries. The model can well explain the data (*R*^2^ > 0.95). We found a strong negative correlation (*r *= − 0.76, *p *< 0.0001, Pearson correlation) between the death rate and the logistic growth rate *k* (Fig. 11e). The death rate is calculated by dividing the number of reported deaths by the number of infected cases. The countries that had more than 200 deaths were selected in this analysis (black dots of Fig. 11e). This finding is in accordance with a general law of virus transmission, which states that the infectivity of a virus is low when its fatality is high. This also implies that *k* is a proxy for intrinsic infectious attributes of a virus.

## Discussion

Based on data for 30 provinces in China from 10 January to 13 April 2020, we have shown that the growth characteristics of infected and recovered cases are positively correlated (Figs. 3b, c). Further, we explained the data from 31 countries using the same model (see methods). We predicted the logistic growth rates (*k*) and semi-saturation times (*t*_0_) of recovered cases based on the knowledge of data from China. There is a strong correlation between these parameters and the timing of governmental emergency policies on COVID-19.

### Comparison with Previous Work

Different from other models that have been applied to COVID-19—such as SIR (Susceptible Infected Recovered Model) or SEIR (Susceptible Exposed Infected Recovered Model) (Li et al. 2020; Tian et al. 2020; Wang, Wang et al. 2020)—our model is relatively simple, but robust. This study is an extension of our previous work.^1^ We have verified that our model explains the data in various geographic spaces of China’s mainland well, and we have achieved robust results with different lengths of time series.

This model is able to capture the macroscopic dynamics of epidemics worldwide (*R*^2^ > 0.95) (Fig. 5), and we quantitatively investigated the minimum data length to produce a stable output (Fig. 4). By April the number of domestic infected cases in China was very limited, which is consistent with our prediction results. The cumulative numbers of infected and recovered cases are almost saturated. This means that the spread of the virus has been effectively controlled. Thus we believe that the parameters estimated in this study for the 30 Chinese provinces are reliable (Fig. 1a–c). The characteristics of two curves can depict the macroscopic features of the epidemic dynamics. On this basis, we applied this model to the data for each of the 31 selected countries (Fig. 5a–c), and found that the sigmoid model fits the data very well too (*R*^2^ > 0.95).

### Relationship Between Model Parameters and the Prediction of Recovered Cases

The semi-saturation periods of infected and recovered cases in China show a positive correlation (*p* = 0.0001). This indicates that the faster the infection cases reach the inflection point, the faster the recovered cases reach their inflection point. We also found a positive correlation between the logistic growth rates of infected and recovered cases (*p* = 0.0359). These results indicate that the increasing rate of recovered cases is strongly correlated with both the increasing patients and their disease control capacity. When comparing *k* and *t*_0_ in specific cases, whether in China’s mainland or countries worldwide (Fig. 8), we found significant negative correlations. This is consistent with our initial work.^1^ With respect to the negative correlation between *k* and *t*_0_ for recovered cases it might also be the case that when the logistic growth rate (*k*) of the recovered cases is larger, it will take less time for most patients to recover.

### Suggestion for Governments Around the World on the Prevention and Control of COVID-19

Most importantly, we found a strong correlation between the timing of governmental control measures for COVID-19 in the 31 selected countries and the semi-saturation period estimated (Fig. 9b–e). This indicates that the early implementation of a government’s prevention and control policy effectively shortened the turning point of the epidemic—assuming consistent implementation of policy measures later on. Taking China as an example, with regard to the COVID-19 outbreak in Wuhan, the strict isolation policy was announced on 23 January, and the inflection point in Wuhan occurred around 9 February 2020, so the time lag was 17 days. Across all countries, the timing of governmental policy is significantly shorter than the semi-saturation period of infected cases, which is also a strong signal for governments of all countries to take control measures as early as possible.

## Conclusion

COVID-2019 outbreaks have brought fundamental changes to the world. While the outbreaks have been controlled in some countries, the further spread is still of great concern in many other countries and regions. To better understand when the pandemic situation may end, we developed a logistic model that can well depict the growth rules of infected and recovered cases in China’s mainland.

The proposed model consists of two key parameters—*k* (the logistic growth rate) and *t*_0_ (the semi-saturation period). This descriptive model has been verified with the infected, death, and recovered cases of COVID-19, and with the SARS data from 2003 in China. Extended results show that this model well explained the data from 31 countries that have been experiencing serious COVID-19 outbreaks (*R*^2^ ≥ 0.95). With the linear relationship between the growth rules for infected and recovered cases identified from the Chinese data, we predicted that the SSP of the recovered cases outside China ranges from 22 March to 8 July. More importantly, there is a strong positive correlation between the SSP of infected cases and the timing of a government’s response. These findings provide strong evidence for the effectiveness of rapid epidemic control measures in various countries.

However, this model is based on a strong assumption that a country or region has relatively consistent epidemic control policies and measures. Given the complex pandemic situation and disaster control practices globally, future study will be extended to model the heterogeneous time series data of cumulative confirmed, dead, and recovered cases with more modeling parameters, in order to further understand the epidemic at a larger spatiotemporal scale.

## Footnotes

1 Han, C.L., Y.M. Liu, and S.N. Yang. 2020. Intrinsic growth rules of patients infected, dead and recovered with 2019 novel coronavirus in mainland China. medRxiv: 2020.02.23.20024802. 10.1101/2020.02.23.20024802.
